# Summaries of "Case" Medicine

**Published:** 1918-10-12

**Authors:** 


### CLINICAL MEMORANDA FOR PRACTITIONERS.

### Summaries of "Case" Medicine.

The most valuable and helpful reference-book on medical practice is the one which is written in the practitioner's individual experience. It is by the stored memories of personal contact with actual events, and of the lessons taught by these events, that is gradually developed the confident and competent medical practitioner. The development of a rich and ready clinical memory is therefore an art greatly to be cultivated. To the fortunate few Nature seems to provide this gift almost ready made. No significant observation escapes them, and the record, once produced, remains as a permanent impression on which they can call at any appropriate moment. But to most men the fates are less kind. Experiences occur, but the memory of them soon becomes vague and uncertain. Their occurrence and the practical values they contain have to be deepened by an effort of the will if they are to live and endure for serviceable ends. It is here that arrives the value of the written record. The practitioner's own clinical note-book, especially if carefully indexed, is a teaching instrument of the highest order, and a confident guide in many dark places. None should neglect its compilation, and necessarily its value and helpfulness increase as the years accumulate. Next to one's own personal experiences come the recorded experiences of others, and this more especially when the records relate to actual facts of observation. Impressions and beliefs and opinions have no doubt their place, but they are of little worth unless we know the facts on which they are based. The general statement is only too often useless in face of a particular difficulty. What the practitioner constantly needs, in short, is a knowledge of "cases." His training in general doctrines is indispensable and may be taken as already in existence. But an increase in his range of efficiency depends essentially upon an increase in his experience of actual clinical events. Necessarily, best of all are those events which happen directly within the area of his own observation. But these may well be seconded by the experiences of others. Many such experiences are reported in our current medical journals, where they are apt to be somewhat overborne by more pretentious communications. We think something may be done to make this body of "case" medicine more generally serviceable, and we propose to make an attempt in this direction by summarising from time to time clinical records which come within our view, and which possess practical values in relation to diagnosis and treatment. For this purpose we shall draw upon our contemporaries, with due acknowledgment, and shall add such comments and references as seem to be appropriate.

### Trench Fever in England.

The problem of unexplained pyrexia is one of the daily difficulties of the practitioner, and the pressure of anxious friends is apt to drive him into a diagnosis which he feels to be premature and uncertain. Of recent years "influenza" has largely offered itself as a convenient retreat, though hardly a scientific one. Now, under the influx of soldiers and others returning from abroad, we may readily be confronted with febrile disturbances of a different order from those commonly met with in home practice. In the *Lancet* of September 21 Dr. F. H. Mosse reports a case of "trench fever" in a lad of eighteen years who had never been abroad and who had only recently joined the Army. The chart showed the characteristic brief pyrexial attacks occurring at intervals of three to eight days over a period of about four weeks. During the febrile stage there was some frontal headache and pain at the back of the eyes, but no vomiting and no catarrhal symptoms; and in the intervals the patient professed himself to be quite well. There were no "shin pains." Influenza and malaria were discussed as possibilities, but were rejected—the former because of the absence of catarrhal symptoms, and the latter in view of the non-periodicity of the fever, the absence of rigors, and the recovery without the administration of quinine. The type of the temperature, with its sharp outbursts of fever separated from one another by periods of apparently complete convalescence, is worthy of note—a combination which has been found useful in relieving the "shin pains" of trench fever is sodium salicylate and tincture of colchicum.

### Jelly-fish Sting Simulating Acute Abdomen.

In the same issue of the *Lancet* Dr. Robert E. Lord and Dr. S. L. B. Willis report an experience of a woman who, stung by a jelly-fish while bathing, was seized with violent abdominal pain and general collapse. The abdominal wall was motionless and exquisitely tender, and here, of course, was the suggestion of some acute abdominal event. Dr. Lord has seen several cases in which, after the sting of a jelly-fish, signs of collapse have been associated with toxic spasm of the muscles of the neck or limbs, and he suggests that the abdominal rigidity in the present record was of this order. The event goes to confirm this, for the patient was able to leave the hospital the day following her admission.

### Emetine Hydrochloride in Hæmophilia.

In the *Practitioner* for September 1918 Dr. J. D. R. Monro relates an impressive experience in the treatment of severe hæmophilia. The patient was a man of twenty-four years, and the family and personal history place the diagnosis entirely beyond dispute. The bleeding on the occasion in question was in the form of hæmaturia. It continued in the most severe form and reduced the patient to a condition from which recovery seemed impossible. Remedies of all sorts, including adrenalin, pituitrin, and horse serum, were tried, but were tried in vain. At last Dr. Monro injected hypodermically ½ grain of emetine hydrochloride. Next morning, though the urine still contained blood, the temperature, which had previously been 104°, had fallen to

**Clinical Memoranda for Practitioners—(*cont.*)**

100°. On the following day the temperature was normal and the urine was unstained, and this after ten weeks of persistent hæmaturia. Of course it may be said that the event was a mere coincidence, and such a possibility cannot be denied. But it stands as a record of observation, and undoubtedly its teaching is that in similar conditions the use of emetine ought to be tried.

### Disappearance of the Evidences of Congenital Cardiac Disease.

The persistence, and sooner or later the fatal issue, of congenital cardiac disease is the common experience, and hence the general inclination is to give an unrelieved prognosis. In the *British Journal of Children's Diseases* for June 1918 Dr. Parkes Weber summarises some experiences which help in some measure to qualify this opinion. He has collected several instances in which such signs as a loud systolic murmur, a thrill, and cyanosis completely disappeared as the patient approached puberty. In one instance the boy at ten years was found eligible for the Navy, and another was passed for military service. Two of the cases rest on the great authority of the late Sir James Goodhart.

### Head Injuries.

Most students, we fancy, are introduced to the generalisation that while no injury to the head is so slight as to be treated lightly, hardly any is so severe as to be inconsistent with recovery. As an illustration of the latter half of the doctrine may be quoted a record made by Dr. T. E. Coulson in the *Lancet* of September 14, 1918. The patient, a boy of nine years, fell from a wagon, both of the wheels of which passed over his head. There were two wounds, from one of which brain matter was oozing, and there was a depressed linear fracture running from before backwards on both sides of the head. After operation the patient's condition remained a very critical one for some forty-eight hours, but he then began to give evidences of consciousness, took food, and showed some degree of control over the bladder and rectum. Ultimately he made a perfect recovery except for slight deafness in one ear.

### The Treatment of Empyema.

When in a case of pneumonia fever persists or returns after a critical fall the practitioner naturally thinks of the probability of empyema, and, if the physical facts justify it, he proceeds to test his suspicion by passing an exploring needle into the chest. If pus is present the question immediately arises whether something more than aspiration—namely, resection of a rib—is necessary. The general rule undoubtedly is that resection should be performed. But in young children at all events, and very occasionally in adults, aspiration proves to be sufficient, for after it the patient makes a perfectly satisfactory recovery.

### Treatment of Locomotor Ataxia.

This disease is apt to make a very hopeless impression on the practitioner's mind, and it must be confessed that many medicinal and other measures which have been advised have failed to justify themselves. Lately, however, it has been shown that in early cases, at all events, good effects may be produced by specific treatment, that is by mercury. The suggestion is that while the actual sclerotic or degenerative changes in the spinal cord cannot be overcome, meningeal and vascular conditions may be, at least in part, improved. It is certain, for example, that such a method often gets rid of the pains which frequently accompany the disease. The same doctrine receives application in a book recently issued by a French physician, Dr. E. Leredde, who claims very good results from the use of arsenobenzene by intravenous injection. He commences with small doses and increases the doses in accordance with the reaction of the patient. After four to seven injections given at intervals of a week or a fortnight the patient is free for a month or so, and then the treatment is repeated. The number of injections required in a series of eighty-seven cases varied from about ten to seventy, and it is noteworthy that good results are claimed even in cases of many years' standing.

### Salicin in Influenza.

Mr. E. B. Turner insists strongly on the value of salicin given in large doses in cases of influenza, and he claims for this medicine prophylactic as well as curative value. He summarises his experience in the *British Medical Journal* of August 3, 1918. In the epidemic of 1891, and in subsequent outbreaks, he ordered twenty grains of salicin every hour and was able to record the most satisfactory results—namely, rapid recovery in the course of a day and a half, and this without complications or sequelæ or a single fatal issue. He has had similar good fortune in the recent outbreak of "Spanish" influenza, while by administering the drug to the non-affected members of the household he has rarely had more than a single patient under one and the same roof.

### Calcium Chloride as a Hæmostatic.

Calcium chloride is often given by the mouth to check hemorrhage, but while this may sometimes be beneficial there are risks about it, for when an excess of calcium exists in the blood the effect is not to increase but to diminish coagulability. Further, pharmacologists say that when calcium chloride is given by the mouth the whole of it is recoverable from the fæces, and hence doubts about the entrance of the salt into the blood cannot be altogether absent. In *Guy's Hospital Gazette*, May 19, 1918, Dr. W. R. Grove advocates administration by intra-muscular injection. He keeps a one-in-four solution of fused calcium chloride and drawing four minims of this into a hypodermic syringe adds boiled water to twenty minims, and he injects the diluted solution deeply into the gluteal muscles. In all cases of hemorrhage where it is impossible to reach the bleeding spot he finds the injection most valuable, and in particular in hæmoptysis it "acts like a charm."

